# ATP utilization by a DEAD-box protein during refolding of a misfolded group I intron ribozyme

**DOI:** 10.1074/jbc.RA120.015029

**Published:** 2020-12-05

**Authors:** Inga Jarmoskaite, Pilar Tijerina, Rick Russell

**Affiliations:** Department of Molecular Biosciences, University of Texas at Austin, Austin, Texas, USA

**Keywords:** ATP, DEAD-box protein, molecular chaperone, nucleic acid structure, ribozyme, RNA, RNA folding, RNA helicase

## Abstract

DEAD-box helicase proteins perform ATP-dependent rearrangements of structured RNAs throughout RNA biology. Short RNA helices are unwound in a single ATPase cycle, but the ATP requirement for more complex RNA structural rearrangements is unknown. Here we measure the amount of ATP used for native refolding of a misfolded group I intron ribozyme by CYT-19, a *Neurospora crassa* DEAD-box protein that functions as a general chaperone for mitochondrial group I introns. By comparing the rates of ATP hydrolysis and ribozyme refolding, we find that several hundred ATP molecules are hydrolyzed during refolding of each ribozyme molecule. After subtracting nonproductive ATP hydrolysis that occurs in the absence of ribozyme refolding, we find that approximately 100 ATPs are hydrolyzed per refolded RNA as a consequence of interactions specific to the misfolded ribozyme. This value is insensitive to changes in ATP and CYT-19 concentration and decreases with decreasing ribozyme stability. Because of earlier findings that ∼90% of global ribozyme unfolding cycles lead back to the kinetically preferred misfolded conformation and are not observed, we estimate that each global unfolding cycle consumes ∼10 ATPs. Our results indicate that CYT-19 functions as a general RNA chaperone by using a stochastic, energy-intensive mechanism to promote RNA unfolding and refolding, suggesting an evolutionary convergence with protein chaperones.

DEAD-box proteins comprise the largest family of RNA helicases and have a broad set of functions throughout RNA biology (1, 2, 3, 4, 5, 6, 7). Many DEAD-box proteins function by promoting rearrangements of structured RNAs, ranging from elements within untranslated regions of mRNAs to folding intermediates in ribosome and spliceosome assembly (6, 8, 9, 10). Despite their functional diversity, DEAD-box proteins share a fundamental activity. They bind double-stranded RNA (dsRNA) and use conformational changes associated with ATP binding, hydrolysis, and nucleotide product release (ADP and P_i_) to separate the two RNA strands from each other and release them sequentially (11, 12, 13, 14, 15, 16, 17, 18, 19). This mechanism leads to local RNA unwinding without processivity or translocation of the protein along the RNA helix (12, 13, 14, 20, 21, 22).

Although we understand the basic mechanism of RNA unwinding by DEAD-box proteins, we have only a limited understanding about how this relatively simple activity is applied to the complex tasks that DEAD-box proteins carry out in biology, including their roles as general RNA chaperones (2, 23, 24, 25). Remodeling of large, structured RNA molecules probably involves both ATP-dependent and ATP-independent steps (26, 27). Indeed, it has been shown that DEAD-box proteins can destabilize RNA tertiary contacts by ATP-independent binding to a dsRNA helical segment that is exposed transiently by spontaneous loss of tertiary contacts, and many DEAD-box proteins possess ATP-independent annealing activity (1, 28). Nevertheless, the efficiencies of DEAD-box proteins in promoting rearrangements of various structured RNAs correlate strongly with their ATP-dependent RNA unwinding activities, suggesting an important role for ATP-dependent RNA unwinding in these processes (29, 30).

General RNA chaperone activity has been studied extensively for the DEAD-box protein CYT-19 (23, 24, 25, 29, 31, 32, 33, 34). CYT-19 promotes splicing of several mitochondrial group I introns in *Neurospora crassa* and can accelerate functional folding of various group I introns and structurally unrelated group II introns *in vitro* and when expressed in *Saccharomyces cerevisiae* (23, 24). Much of the knowledge about the chaperone mechanism of CYT-19 has been obtained through *in vitro* studies using the well-characterized group I intron ribozyme from *Tetrahymena thermophila* (Fig. 1*A*) (23, 25, 31, 32, 33, 34). The ribozyme consists of a conserved catalytic core and several peripheral domains, and it catalyzes oligonucleotide cleavage reactions analogous to the two transesterification steps of group I intron splicing (35, 36, 37, 38). After folding is initiated with Mg^2+^ ions *in vitro*, the ribozyme folds predominantly to an inactive, misfolded conformation, which then slowly refolds to the thermodynamically favored native state (39, 40). This long-lived misfolded structure is structurally similar to the native structure, including all of the native secondary structure elements and five long-range tertiary contacts (41). The origin of misfolding appears to be topological, with two single-stranded elements being incorrectly crossed in the center of the ribozyme (41, 42). Because this topological error is surrounded by a network of native interactions, refolding to the native state requires extensive unfolding that includes disruption of all five tertiary contacts and the P3 helix in the ribozyme core (41, 42).

**Figure 1:**
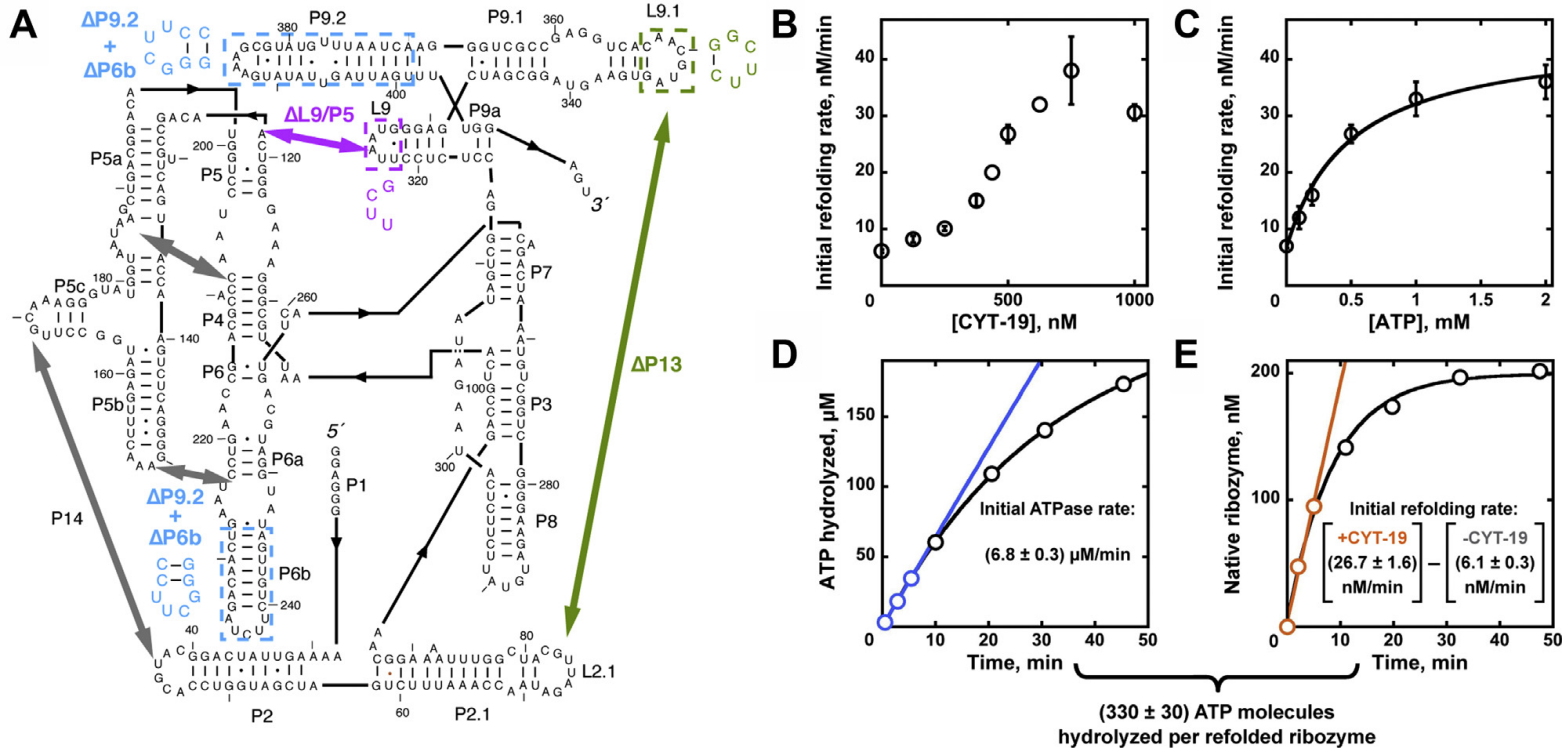
**CYT-19–mediated refolding of the misfolded *Tetrahymena* ribozyme and ribozyme-dependent ATPase activity.***A*, ribozyme secondary structure and long-range tertiary contacts. *Thick arrows* indicate tertiary contacts. L, loop; P, paired region. Mutants used here are shown in color. The ΔP13 (*green*) and ΔL9/P5 (*magenta*) mutants lack the indicated tertiary contact as a result of the sequence changes indicated. The ΔP9.2 + ΔP6b mutant has the two indicated helices truncated. *B*–*C*, CYT-19 and ATP concentration dependences of the refolding rate from the misfolded state to the native state. All measurements were performed with 200 nM misfolded ribozyme at 2 mM Mg(OAc)_2_ (pH 7.0, 25 °C). CYT-19 concentration was varied in the presence of 500 μM ATP (in all cases, ATP was added as ATP-Mg^2+^), and ATP concentration was varied in the presence of 500 nM CYT-19. Averages and standard errors from 2 to 10 determinations are shown, except for single measurements at 440 nM and 625 nM CYT-19 in panel B; see Tables S1 and S2. The upward curvature in panel B arises from RNA binding and titration of CYT-19 (see Fig. S1). *D*–*E*, initial rates of the ATPase and ribozyme refolding activities of CYT-19. Representative data with 500 nM CYT-19 and 500 μM ATP are shown, and the values reflect averages and standard errors from 10 (ATPase and CYT-19–dependent refolding) and 2 (spontaneous refolding) determinations. The refolding curves in panel *E* were normalized to the total ribozyme concentration (200 nM). The value at the bottom indicates the ATP utilization per refolded ribozyme as the initial ATPase rate divided by the initial rate of refolding, with the spontaneous refolding rate subtracted.

Although spontaneous refolding is extremely slow, on the time scale of hours, CYT-19 accelerates this transition in an ATP-dependent manner by disrupting the misfolded structure (25, 31). CYT-19 unfolds the misfolded intermediate much more readily than the native ribozyme, apparently because the misfolded structure is less stable (25). Consistent with the role of RNA structural stability in controlling the CYT-19–mediated unfolding rate, CYT-19 can also efficiently disrupt the native ribozyme if it is destabilized by mutations (25, 33). Studies with ribozyme variants that vary in local or global stability suggest that CYT-19 preferentially interacts with exposed RNA helices, which are present more frequently in RNAs with a weakened tertiary structure (28, 31, 33). Thus, CYT-19 is suggested to unfold the ribozyme structure by capturing and unwinding ribozyme helices that are transiently exposed by spontaneous losses of tertiary structure. However, it is unknown how many such local disruptions and ATP-dependent steps are required for unfolding. It is also unknown whether global disruption of the ribozyme structure is achieved *via* a well-defined sequence of steps or a multitude of pathways that may involve predominantly spurious interactions of CYT-19 with the RNA.

Here we probe the RNA chaperone mechanism of CYT-19 by measuring the number of ATP molecules that are hydrolyzed during native refolding of the misfolded ribozyme. We show that hundreds of ATP molecules are hydrolyzed for every ribozyme molecule that reaches the native state. Further dissection of this value suggests that most of the ATPase activity arises from nonproductive RNA unwinding events that are unlinked to the folding transition, as well as from futile cycles of unfolding that result in reformation of the misfolded structure. We suggest that the energy-intensive nature of the refolding activity is inherent to chaperones that function with broad substrate specificity, both RNA chaperones and protein chaperones.

## Results

### Measuring ATP utilization for refolding of the misfolded ribozyme

To determine the amount of ATP consumed by CYT-19 during native refolding of the misfolded *Tetrahymena* ribozyme (the ATP utilization value), we first surveyed conditions to maximize the CYT-19–dependent and ATP-dependent acceleration of refolding above the spontaneous rate, such that as much as possible of the observed refolding could be attributed to CYT-19 activity. To measure the refolding rates, we used a previously established two-stage folding assay (43, 44). The ribozyme was first misfolded by a brief incubation with 10 mM Mg^2+^, and then refolding was initiated by dilution of Mg^2+^ to 2 mM and simultaneous addition of CYT-19 and ATP (stage 1). At various time points, the folding reaction was stopped by increasing the Mg^2+^ concentration and adding proteinase K. The fraction of the native ribozyme present at each time point was determined by cleavage of a short radiolabeled oligonucleotide substrate (CCCUCUA_5_) that base pairs with the 5′ end of the ribozyme, mimicking the 5′ splice site of the natural self-splicing reaction (stage 2). Because the substrate binds tightly and with comparable rate constants to both the native and misfolded conformations, the fraction of the substrate that is cleaved rapidly by the native ribozyme reports on the fraction of the ribozyme that is present in the native state. By measuring the refolding rates at several CYT-19 and ATP concentrations, we found that the refolding rate was increased up to ∼6-fold at 2 mM Mg^2+^, 200 nM ribozyme, ≥1 mM ATP, and ≥500 nM CYT-19 at 25 °C (Fig. 1, *B*–*C*). Increasing the CYT-19 concentration further did not increase the refolding rate.

To determine the number of ATPs hydrolyzed per refolded ribozyme, we measured the ATPase rate during refolding at concentrations of CYT-19 (500 nM) and ATP (500 μM) close to saturation, corresponding to a 4.4-fold acceleration of refolding (Fig. 1, *B*–*C*). The ATP utilization value was then determined by dividing the initial rate of ATP hydrolysis by the initial rate of CYT-19–mediated ribozyme refolding, yielding 330 ATP molecules per ribozyme molecule (Fig. 1, *D*–*E*). As demonstrated in the subsequent sections, changes in the ribozyme and CYT-19 concentrations did not strongly affect the ATP utilization.

The ATP utilization value of 330 likely includes both productive and futile ATP hydrolysis events, *i.e.*, ATP hydrolysis events that contribute to misfolded ribozyme unfolding and some that do not contribute to ribozyme unfolding. Previous measurements revealed robust stimulation of the ATPase activity of CYT-19 by the native ribozyme, despite a lack of detectable unfolding (33). Much of this nonproductive ATPase activity arises from interactions of CYT-19 with two helices that protrude from the folded structure, as truncations substantially reduced the ATPase stimulation (ΔP6b+ΔP9.2; see Fig. 1*A*) (33). These helices are formed in the misfolded structure and are expected to be similarly accessible, providing a likely source of futile ATP hydrolysis events (41). To test the alternative possibility that CYT-19 interactions with these helices can be productive for misfolded ribozyme unfolding, we measured CYT-19–dependent refolding of the truncation mutant ΔP6b+ΔP9.2 (Fig. S1). We found that refolding of the mutant was at least as fast as refolding of the wild-type ribozyme, providing no evidence for a productive role of these helices in refolding.

In light of these results, we reasoned that the ATPase activity with the native ribozyme provides an estimate of the ‘background’ ATPase activity that is not associated with productive unfolding. A comparison of the initial ATPase rates measured with the misfolded or native ribozyme under identical conditions revealed a 50% enhancement of ATPase activity in the presence of the misfolded ribozyme [(6.8 ± 0.3) μM/min *versus* (4.5 ± 0.3) μM/min; Table S1], most simply suggesting that approximately two-thirds of the ATP hydrolysis events with the misfolded ribozyme result from nonproductive interactions with constitutively accessible regions, with the remaining one-third occurring during unfolding of the misfolded RNA. This latter value corresponds to approximately 110 ATP molecules hydrolyzed per ribozyme molecule refolded.

### Concordance of ATP hydrolysis and ribozyme refolding kinetics

**Figure 2:**
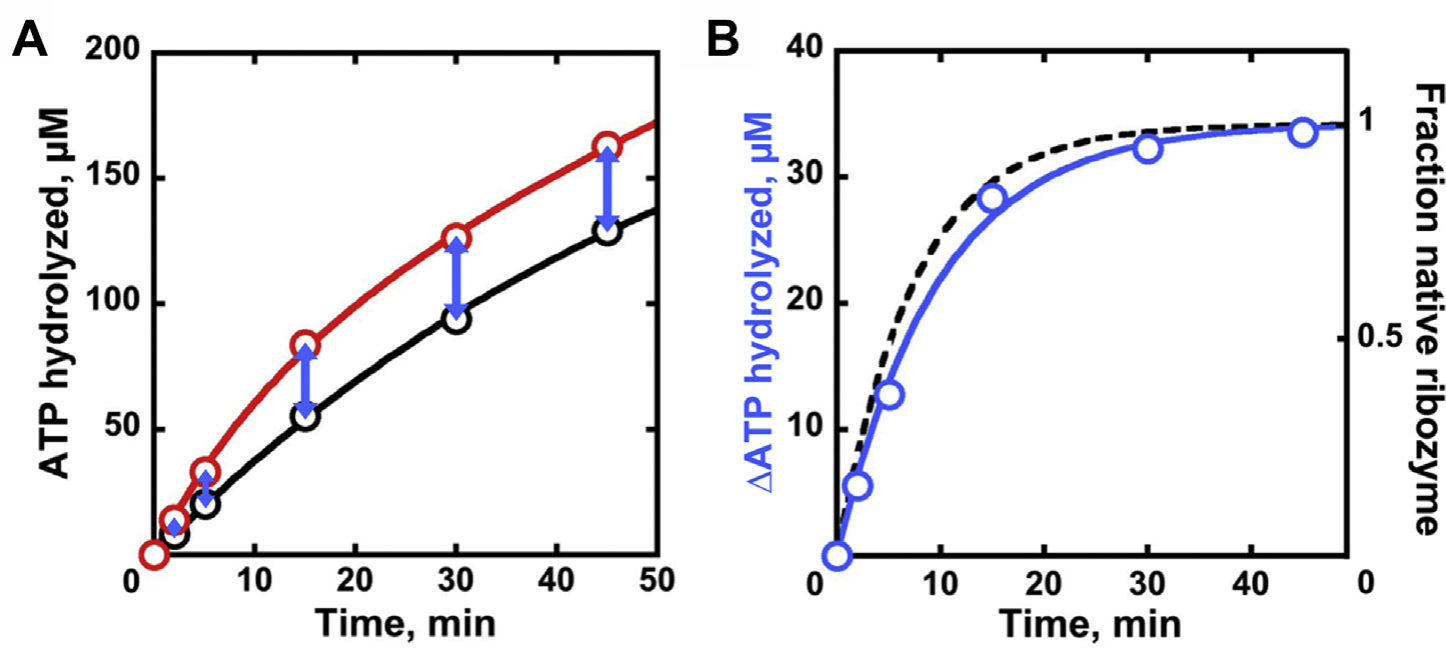
**ATP hydrolysis during the refolding reaction.***A*, ATPase activity monitored over the complete time course of refolding of the misfolded ribozyme (*red*). ATPase activity with the preformed native ribozyme (measured in parallel) is shown in *black*. Representative time courses for 500 nM CYT-19 and 500 μM ATP-Mg^2+^ are shown (200 nM ribozyme, 2 mM Mg^2+^). *B*, *blue circles* represent the additional ATP hydrolyzed (‘ΔATP’) at each time point in the refolding reaction compared with an analogous reaction in the presence of the native ribozyme (*blue arrows* in part *A*). The resulting curve can be fit by a single-exponential equation, with the rate constant equivalent to that of refolding measured under the same conditions by ribozyme catalytic activity (*dashed curve*; see Fig. 1*B*). The amplitude of the *blue curve* corresponds to the amount of ATP hydrolyzed during CYT-19 interactions unique to the misfolded ribozyme. Dividing this value by the ribozyme concentration (200 nM) gives the number of ATP molecules used for misfolded ribozyme refolding.

If the increased CYT-19–dependent ATP hydrolysis rate observed with the misfolded ribozyme resulted from CYT-19–mediated ribozyme unfolding, we would expect the ATPase rate to decrease over the time course of the refolding reaction, eventually reaching the lower level of stimulation characteristic of the native ribozyme. To test this prediction, we initiated refolding of the misfolded ribozyme and monitored ATP hydrolysis over a time sufficient for complete refolding, as determined by the ribozyme catalytic activity assay. In parallel, we measured the ATPase activity in a reaction in which the ribozyme was prefolded to the native state. The data revealed rapid ATPase activity in the presence of the misfolded ribozyme, which slowed over time until it matched the rate measured with the native ribozyme (Fig. 2*A*). Some curvature was also present in the ATPase time course obtained with the native ribozyme, most likely due to ATP depletion and inhibition by accumulated ADP (45), but it was much less pronounced. A plot of the difference between the amounts of ATP hydrolyzed in the two reactions gave a single-exponential curve with a rate constant that was indistinguishable from the CYT-19–dependent refolding rate constant measured by ribozyme activity under the same conditions. This result demonstrates that the enhancement in ATPase activity reflects ATP hydrolysis during ribozyme refolding. Furthermore, the amplitude of the differential activity curve provides a direct readout of the amount of ATP consumed for unfolding of the misfolded ribozyme. At the conditions tested above (500 nM CYT-19, 500 μM ATP), this value was 160 ± 10 ATP molecules per misfolded ribozyme, in reasonable agreement with the value of 110 ATPs calculated from the initial rates in the previous section.

### Consistent ATP utilization across CYT-19 and ATP concentrations

**Figure 3:**
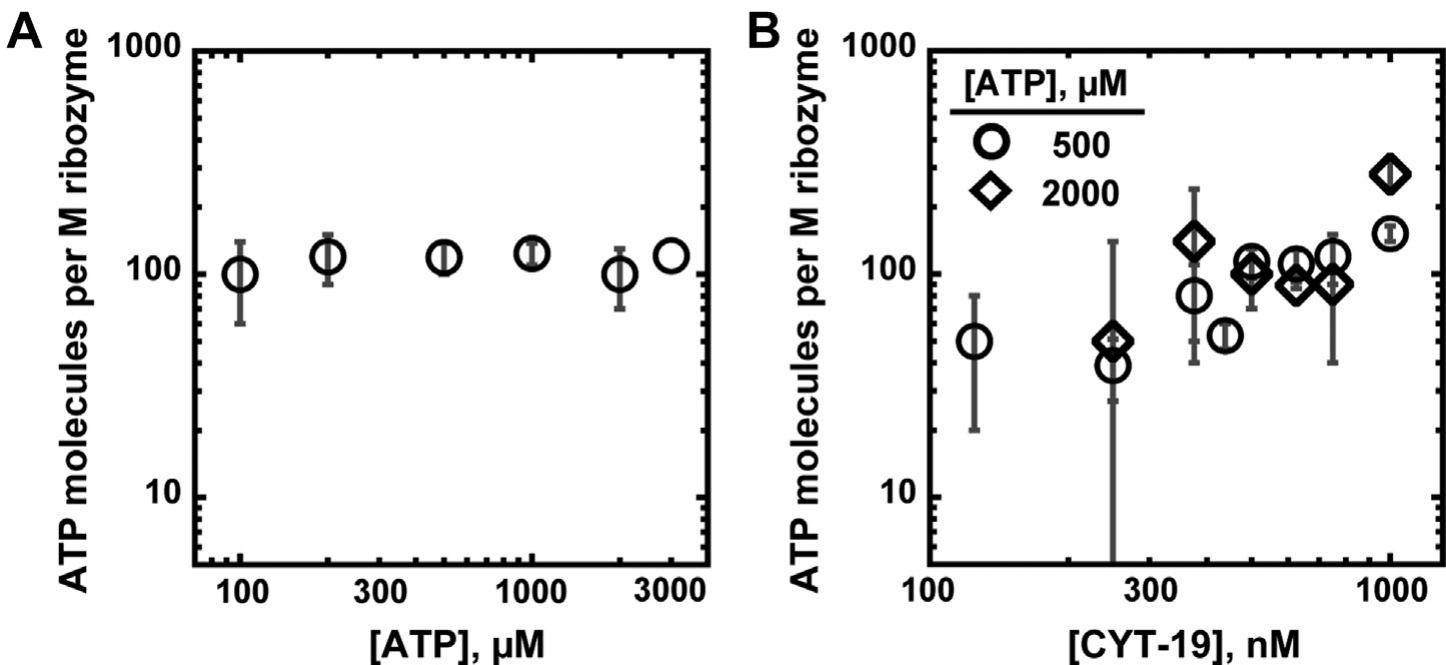
**ATP utilization across ATP and CYT-19 concentrations.***A*, ATP concentration dependence of the ATP utilization value (500 nM CYT-19, 200 nM ribozyme). Averages and standard errors are shown; see Table S1. *B*, CYT-19 concentration dependence of ATP utilization in the presence of 500 μM ATP (*circles*) or 2 mM ATP (*diamonds*). Averages and standard errors are shown; see Tables S2 and S3.

We next measured how the ATP utilization value depends on the CYT-19 and ATP concentrations. At each condition, we determined the level of ATP utilization from the difference between initial ATPase rates measured with the misfolded or native ribozymes, divided by the initial rate of CYT-19–dependent refolding under identical conditions. Varying the ATP concentration across a 30-fold range at a constant CYT-19 concentration (500 nM) revealed a uniform ATP utilization value of approximately 100 ATP molecules per ribozyme (Fig. 3*A*). Varying the CYT-19 concentration at 500 μM or 2 mM ATP revealed little change, with at most a slight increase in ATP consumption with increasing CYT-19 concentration (Fig. 3*B*). Thus, our results suggest that the ATP efficiency of CYT-19 activity does not improve with increasing CYT-19 or ATP concentrations. These observations suggest that increases in nonproductive ATP-hydrolysis events at higher CYT-19 and ATP concentrations balance or even exceed any potential gains in efficiency of ATP utilization from decreasing the number of futile cycles. The results also reveal a relatively well-constrained ATP utilization value of ∼100 ATP molecules across a range of conditions.

### Increase in ATP utilization with increasing ribozyme stability

Prior measurements of ATP utilization for RNA helix unwinding revealed increased ATP consumption for unwinding of more stable helices, most likely because of an increase in futile ATPase cycles that do not result in helix unwinding (13). To test whether the stability of the misfolded ribozyme structure similarly affected the ATP requirement for ribozyme refolding, we varied the stability of the misfolded ribozyme by varying the magnesium concentration or by introducing destabilizing mutations (40, 41).

**Figure 4:**
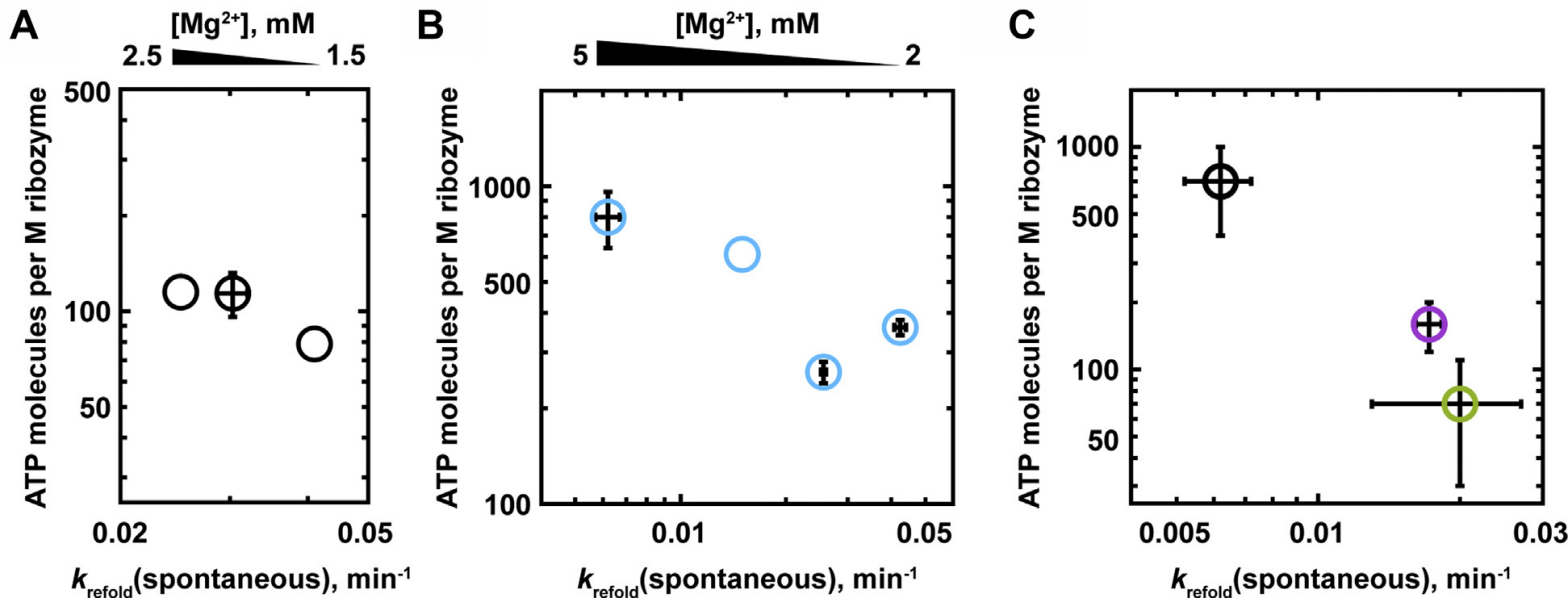
**Effects of ribozyme stability on ATP utilization for refolding.***A*, Mg^2+^ concentration dependence of ATP utilization for refolding of the misfolded WT ribozyme (500 nM CYT-19, 500 μM ATP-Mg^2+^). ATP utilization values were derived from initial ATPase and refolding rates; see Table S4. The ATP utilization values are plotted against the rate constants of spontaneous refolding, which provide a measure of misfolded ribozyme stability, as they reflect the energetic difference between the misfolded ribozyme and the less structured transition-state ensembles for refolding to the native structure. Values correspond to single measurements, with the exception of 2 mM Mg^2+^, where averages and standard errors from 10 measurements are shown. *B*, Mg^2+^ concentration dependence of ATP utilization for refolding of the misfolded ΔP9.2+ΔP6b ribozyme. Averages and standard errors from two determinations are shown, except for single determinations at 4 mM Mg^2+^. Reactions included 200 nM misfolded ribozyme, 1 μM CYT-19, and 2 mM ATP-Mg^2+^; see Table S5. *C*, ATP utilization for refolding of misfolded wild-type and tertiary contact mutant ribozymes. Data are shown for the wild-type ribozyme (*black*) and two mutants with tertiary contact disruptions (ΔL9/P5, *magenta*; ΔP13, *green*; see Fig. 1*A*). Reactions included 200 nM ribozyme, 500 nM CYT-19, and 2 mM ATP-Mg^2+^ at 5 mM Mg^2+^. Averages and standard errors from 2 to 3 experiments are shown. See Table S6.

For the wild-type ribozyme, varying the Mg^2+^ concentration from 1.5 to 2.5 mM did not change the ATP utilization significantly (Fig. 4*A*; Table S4). The accessible range of Mg^2+^ concentrations was limited because at lower Mg^2+^ concentrations, the native ribozyme became unstable enough to also be unfolded by CYT-19 (25), and at higher Mg^2+^ concentrations, the enhancement in ATPase stimulation by the misfolded ribozyme relative to that for the native ribozyme became too small to measure (Figs. S2 and S3).

To extend the measurable Mg^2+^ concentration range, we aimed to reduce the background ATPase activity by using the helix-truncated mutant ΔP6b+ΔP9.2 (see Fig. 1*A*). These measurements were made in the presence of a higher CYT-19 concentration (1 μM *versus* 500 nM above) and ATP concentration (2 mM *versus* 500 μM above), which moderately increased the ATP utilization values and the robustness of the CYT-19–mediated refolding. Under these conditions, we were able to collect reliable measurements from 2 to 5 mM Mg^2+^. The spontaneous refolding rate was decreased by 6.9-fold with increasing Mg^2+^ concentration across this range, and the ATP utilization value increased, albeit by a smaller amount, 2.2-fold (Fig. 4*B*; Table S5). Overall the Mg^2+^ dependence data suggest that there are a relatively well-constrained number of ATP-dependent steps in CYT-19–mediated unfolding of the misfolded ribozyme and that increasing the stability of the misfolded ribozyme modestly increases the ATP requirement.

As an orthogonal approach to varying the misfolded ribozyme stability, we compared the wild-type ribozyme with two ribozyme mutants that have disruptions of long-range tertiary contacts and were shown previously to form destabilized misfolded structures (Fig. 1*A*) (41). To prevent CYT-19–mediated unfolding of the native ribozyme mutants, these measurements were performed at 5 mM Mg^2+^. The misfolded conformations of the mutants were approximately 3-fold less stable than those of the wild-type ribozyme, as determined by their intrinsic refolding rates, and both mutants gave ATP requirements approximately 5- to 10-fold smaller than that of the wild-type ribozyme (Fig. 4*C*, Table S6). Thus, under identical conditions, CYT-19–mediated refolding of less stable misfolded ribozyme variants requires less ATP, suggesting fewer ATP-dependent steps. The reduced ATP requirement for unfolding of the destabilized mutants may arise from a greater contribution of spontaneous events to global unfolding or from a difference in the unfolding pathway, most simply a result of fewer tertiary contacts that must be disrupted for the mutants.

## Discussion

The ability of DEAD-box helicases to promote ATP-dependent RNA folding transitions is well established, but we have relatively little information about the steps within these complex processes. Here we quantified ATP utilization for refolding of the misfolded group I intron ribozyme by the DEAD-box protein CYT-19, revealing that hundreds of ATP molecules are consumed during each refolding event. The data also suggest that productive rearrangements result from only a minor fraction of total ATP utilization. Below we discuss potential sources of futile and productive ATP hydrolysis during CYT-19–mediated refolding.

It is useful to consider the sources of ATP consumption in the context of what we already know about the structure of the misfolded ribozyme and the mechanism of refolding. The only secondary structure element that is known to be disrupted during refolding is the P3 helix (see Fig. 1*A*), which, if unwound directly by CYT-19 in the same manner as an isolated RNA helix, would be expected to require hydrolysis of a single ATP molecule (13, 41, 42). All five peripheral tertiary contacts are also known to be transiently disrupted during refolding (41). Lacking a mechanism to disrupt tertiary structure directly, CYT-19 is able to bind and trap helices that lose tertiary contacts spontaneously in an ATP-independent manner (28, 33). It also remains possible that CYT-19 can disrupt tertiary contacts through unwinding of adjacent helices (28, 41). Even if one or two helices must be unwound to promote disruption of each tertiary contact, and all five contacts are disrupted actively by CYT-19, one would expect 5 to 10 ATP molecules to be used for global unfolding of the tertiary structure. In contrast, our observations of hundreds of ATP molecules hydrolyzed per refolding event dwarfs the numbers from these parsimonious models.

**Figure 5:**
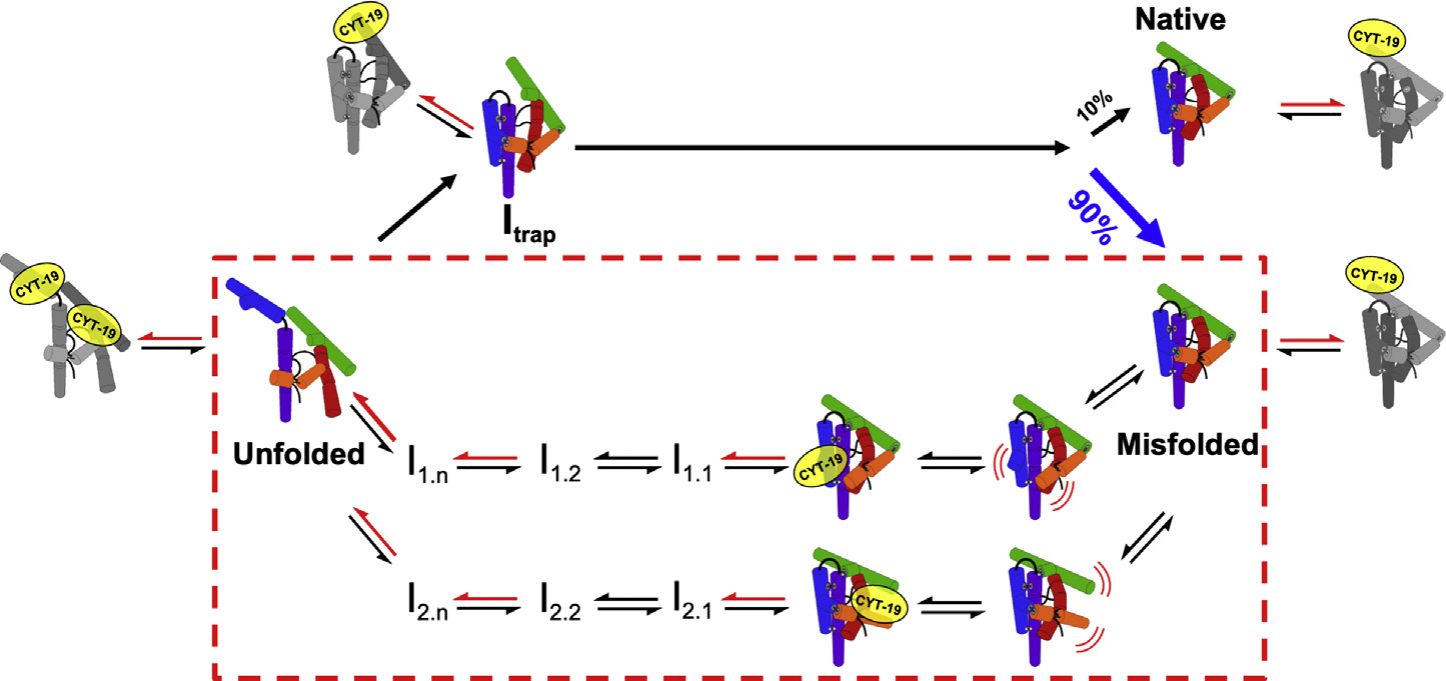
**Model for CYT-19-mediated refolding of the misfolded *Tetrahymena* ribozyme.** Following a spontaneous local unfolding event in the misfolded ribozyme structure (right), CYT-19 binds and unwinds an exposed RNA helix, promoting further unfolding through a series of intermediates and ATP-dependent (*red arrows*) and ATP-independent (*black arrows*) steps. The two parallel pathways illustrate that unfolding can most likely initiate from one of several sites, probably determined stochastically by local accessibility of RNA helices. Folding to the native state is essentially irreversible under these conditions (25). Futile interactions, depicted as gray ribozyme schematics with bound CYT-19, indicate CYT-19 interactions that consume ATP but do not yield rearrangements that are productive for global unfolding.

Together with the understanding of the ribozyme folding pathway from earlier work, our results suggest several origins of the high ATP utilization value and lead to the model of CYT-19-dependent unfolding of the misfolded ribozyme and subsequent refolding shown in Figure 5. Starting from the misfolded ribozyme, a substantial fraction of the ATP hydrolysis events likely reflect spurious interactions with protruding helices that do not result in unfolding (Fig. 5, far right bottom). These interactions are mirrored for the native ribozyme (Fig. 5, far right top), producing a ‘background’ of ATP hydrolysis that amounts to approximately two-thirds of the total observed with the misfolded ribozyme under conditions that approximate physiological Mg^2+^ concentrations. Upon subtraction of this background activity, it is revealed that unfolding and refolding of each ribozyme molecule is accompanied by hydrolysis of approximately 100 ATP molecules. It should be noted that while we consider this value to be the best estimate of the ATP consumption during refolding, it likely represents a lower limit, as our subtraction-based approach assumes that all interactions formed with the native ribozyme are formed with the misfolded ribozyme with the same frequency and are nonproductive for refolding. Omitting the background subtraction completely gives a value of ∼300 ATPs per refolding event as an upper limit (Fig. 1, *D*–*E*). In a plausible intermediate scenario, nonproductive background ATPase activity arises exclusively from CYT-19 interactions with the protruding helices P9.2, P6b, and P8 (which collectively amount to ∼50% of ATPase activity with the native ribozyme (33)). Subtracting only the background ATPase activity traceable to these helices, instead of all ATPase activity with the native ribozyme, leads to an intermediate ATP utilization value of ∼200 ATPs.

Nevertheless, even the lower limit of 100 ATPs is substantially more than are likely to be required for unfolding of the misfolded ribozyme. We suggest that reversibility of the unfolding steps, at small and large scales, likely accounts for a large fraction of this ATP usage. Once unfolding of the misfolded ribozyme is initiated (right to left), most likely through a spontaneous opening of one of the peripheral tertiary contacts, new potential interaction sites become accessible to CYT-19 (28, 31, 33). What follows is likely a combination of spontaneous unfolding steps and CYT-19–dependent steps, probably along multiple unfolding pathways (depicted schematically in Fig. 5 by the presence of two parallel pathways). Unfolding can be spontaneously reversed at any point along these unfolding pathways. The implication for ATP utilization is that some or all of the ATP-dependent steps may need to be repeated multiple times before the misfolded ribozyme is unfolded sufficiently that it can enter a pathway that can progress to the native state (Fig. 5, transition from bottom to top).

In addition to this local reversibility, studies of ribozyme folding have provided evidence for larger scale reversibility. During ‘intrinsic’ folding of the wild-type ribozyme and the mutants used herein, the ribozyme reaches a branchpoint late in the folding process from which it is ∼10-fold more likely to misfold than to fold correctly (Fig. 5) (39, 40, 46). Consequently, the simplest expectation is that the misfolded ribozyme needs to be globally unfolded approximately 10 times, on average, before it reaches the native state. Thus, each global unfolding cycle of a misfolded ribozyme by CYT-19 likely requires only 10% of the total ∼100 ATP molecules (10 ATPs), suggesting that there are 10 ATP-dependent steps in each global unfolding cycle. These 10 steps may consist of 10 different productive steps, 10 repetitions of a single step (*e.g.*, unwinding of the P3 helix), or a combination of productive and nonproductive steps and their repetitions.

It is notable that the high propensity of this intron to misfold is likely to contribute substantially to the ATP requirement. It is not clear whether this misfolded conformation forms during intron transcription and folding *in vivo*. Native state folding, as measured by the splicing rate, appears to be much more efficient *in vivo* than *in vitro* (47, 48), but it is not known whether this increase reflects avoidance of the misfolded conformation or its resolution by endogenous chaperones. More generally, it is possible that long-lived misfolded conformations of certain functional RNAs could be advantageous by providing a pool of material primed for rapid activation by DEAD-box proteins.

Analogous ATP utilization values for other DEAD-box proteins are likely to vary considerably depending on RNA structure, the type of rearrangement, and the specific protein involved. At one extreme, DEAD-box proteins that are specifically recruited to individual RNA or RNP substrates and promote well-defined structural rearrangements may consume much less ATP than CYT-19. At the other extreme, other DEAD-box proteins that possess general chaperone activity and interact with diverse RNAs, such as Ded1 and Mss116 in yeast and CsdA in bacteria, likely also consume substantial ATP due to nonproductive interactions (4, 24, 49). Factors that limit such indiscriminate activity *in vivo* may include compact native RNA folding and decay of misfolded or damaged RNAs that lack compact structures or protein interaction partners. The consumption of 100 ATPs during refolding of the misfolded *Tetrahymena* ribozyme may be toward the high end for RNA refolding events because of the requirement for large-scale unfolding and the tendency for repeated misfolding.

In summary, we show that hundreds of ATP molecules are hydrolyzed during refolding of a misfolded ribozyme by the DEAD-box helicase CYT-19. Furthermore, we identify sources of nonproductive ATP hydrolysis that contribute to this high ATP consumption, narrowing down the number of productive ATP-dependent steps to approximately 10. Given the requirement for extensive unfolding during refolding of the misfolded ribozyme, it is likely that CYT-19 disrupts multiple helices in the course of unfolding by interacting stochastically with helices that become exposed by spontaneous disruptions of the tertiary structure or during prior steps of CYT-19–assisted unfolding. Further work will be required to determine which structural elements are actively disrupted by CYT-19 and whether unfolding proceeds along a defined set of pathways or *via* many different pathways.

The high ATP requirement exhibited by CYT-19 is likely to be characteristic of general chaperones. Indeed, high energetic costs of promoting structural rearrangements have also been found for several protein chaperones. The GroEL-GroES chaperonin hydrolyzes 14 ATP molecules per cycle of protein encapsulation, and multiple cycles are typically required for refolding of a misfolded protein, with ATP utilization values of 100 or more reported per refolded protein monomer (50, 51, 52). ClpX, the unfoldase component of the bacterial ClpXP protease, requires several hundred ATP molecules to unfold a domain of the titin protein with an attached degradation tag, with a lower ATP requirement for less stable substrates (53, 54, 55). Most of the ATP appears to be consumed by ClpX during futile attempts to capture the degradation tag sequence, which is required for initiation of unfolding (56). For the Hsp70 chaperone system, ATP utilization varies from 5 ATPs per misfolded luciferase to more than 1000 ATPs for refolding of stable glucose-6-phosphate dehydrogenase aggregates (57, 58).

In light of our results, both protein and RNA chaperones appear to have evolved to use stochastic, energy-intensive mechanisms to promote native folding by accelerating unfolding processes. This mechanism is probably imposed by their functions. Because general chaperones must be able to engage with diverse structures, it is difficult to imagine how they could avoid nonproductive interactions with native macromolecules and with structural modules or domains that do not lead to productive unfolding reactions. In addition, it is likely that the high (mM) cellular ATP levels and the fact that ATP consumption by cellular processes like protein synthesis greatly exceeds that of protein and RNA refolding lead to an absence of selective pressure for lower ATP utilization by general chaperones (59, 60, 61). The functional convergence of this mechanism in protein and RNA chaperones suggests that it is an efficient solution to the general problem of maximizing macromolecular folding to functional structures in the cellular context.

## Experimental procedures

### Materials

The L-21/ScaI form of the *Tetrahymena* ribozyme (wild-type and mutants shown in Fig. 1*A*) was transcribed by T7 RNA polymerase *in vitro* and purified using Qiagen RNeasy columns as described (33, 39). Ribozyme concentrations were determined by absorbance at 260 nm (extinction coefficient of 3.9 × 10^6^ M^−1^ cm^−1^ for the wild-type ribozyme and tertiary-contact mutants, and 3.6 × 10^6^ M^−1^ cm^−1^ for the ΔP9.2+ΔP6b mutant). The oligonucleotide substrate rSA_5_ (CCCUCUA_5_) was 5ʹ-labeled with [γ-^32^P]ATP (Perkin-Elmer) using T4 polynucleotide kinase, purified by denaturing PAGE [20%; 7 M urea, TBE buffer (89 mM Tris-borate, 2 mM EDTA, pH 8.3)], and stored in TE buffer (10 mM Tris-Cl, pH 8.0, and 1 mM EDTA) at −20 °C (62). CYT-19 was expressed and purified as described (28), and the protein concentration was determined *via* Bradford assay with bovine serum albumin used as a standard.

### Ribozyme refolding measurements

Refolding of the misfolded ribozyme was monitored by using ribozyme catalytic activity as a readout for native state formation (43, 44). The misfolded ribozyme was prepared by incubating 2 μM ribozyme with 10 mM Mg(OAc)_2_ and 50 mM Na-MOPS (pH 7.0) for 5 min at 25 °C and stored on ice until the measurement. Refolding reactions were initiated by addition of the ribozyme (2 μl) and CYT-19 (or an equivalent volume of CYT-19 storage buffer [20 mM Tris-Cl (pH 7.5), 500 mM KCl, 1 mM EDTA, 0.2 mM DTT, and 50% glycerol (vol/vol)]; 2 μl) to a reaction mix (16 μl) containing the desired concentrations of Mg(OAc)_2_ and ATP-Mg^2+^ in 50 mM Na-MOPS (pH 7.0). Refolding was monitored at 25 °C; at varying time points, reaction aliquots (2 μl) were transferred to a folding quench solution (4 μl; 72.5–74 mM MgCl_2_ (for a final concentration of 50 mM Mg^2+^), 750 μM guanosine (500 μM final), 1.5 mg/ml proteinase K and 50 mM Na-Mops (pH 7.0)). The high magnesium concentration effectively stops folding by trapping all ribozymes in the long-lived misfolded and native states and, along with guanosine, creates the necessary conditions for the subsequent catalytic step, while proteinase K degrades CYT-19. To determine the fraction of the native ribozyme, after ≥2 min at room temperature, 1 μl of trace radiolabeled rSA_5_ (∼1 nM) was added, and the burst amplitude of substrate cleavage activity was measured by stopping the cleavage reaction after 1 min with 2-fold excess of an EDTA-containing gel-loading buffer (72% formamide (vol/vol), 100 mM EDTA, 0.4 mg/ml xylene cyanol, and 0.4 mg/ml bromophenol blue) and determining the fraction of cleaved rSA_5_ by 20% denaturing PAGE (40). The burst amplitude reflects the fraction of the native ribozyme because both the misfolded and native ribozymes can bind the substrate at the same rate, but only the native ribozyme can cleave the substrate. The large excess of ribozyme over substrate and the slow dissociation of the uncleaved substrate from the misfolded ribozyme ensure single-turnover cleavage conditions with a well-defined burst (43, 44).

### ATPase measurements

ATPase rate measurements were performed under conditions identical to those of the refolding reactions, except for the addition of trace [γ-^32^P]ATP. Misfolded ribozyme was prepared as above (5 min at 10 mM Mg(OAc)_2_, 25 °C), and native ribozyme was formed by a 45-min incubation at 50 °C, 10 mM Mg(OAc)_2_ (50 mM Na-MOPS, pH 7.0), followed by storage on ice. At varying time points after mixing with CYT-19 and ATP-Mg^2+^, aliquots (2 μl) were quenched by mixing with 150 mM EDTA (4 μl). Measurements with native and misfolded ribozyme were performed in parallel. The hydrolysis products were separated by thin-layer chromatography (1 M formic acid, 0.5 M LiCl) and quantified using a phosphorimager and ImageQuant. The small amounts of contaminating ^32^P_i_ present in the [γ-^32^P]ATP stocks (0.4–2.3%) were subtracted, and the fraction of hydrolyzed ATP was multiplied by the total ATP concentration to determine the ATPase rates. To control for the background ATPase activity of CYT-19, including any stimulation that could result from small amounts of RNA that may be copurified with CYT-19, the ATPase activity was also measured in the absence of added ribozyme. The observed ATPase rates were consistently at least an order of magnitude below those measured with added ribozyme under identical conditions (Table S7). Importantly, the subtraction-based method of quantifying ATP utilization corrects for this low level of ribozyme-independent ATPase activity, as it is expected to contribute equally to the ATPase activity in the presence of the misfolded or the native ribozyme.

## Data availability

Original data are available upon request (Rick Russell; rick_russell@cm.utexas.edu).

## Conflict of interest

The authors declare that they have no conflicts of interest with the contents of this article.
