# Supplementary material for: ATP utilization by a DEAD-box protein during refolding of a misfolded group I intron ribozyme
Source: J Biol Chem. 2020 Dec 5;296:100132. doi: 10.1074/jbc.RA120.015029 (PMC7948464; doi:10.1074/jbc.RA120.015029)
Supplement: Supplementary Figures and Tables [file mmc1.pdf]

ATP utilization by a DEAD-box protein during refolding of a misfolded group I intron ribozyme

Inga Jarmoskaite, Pilar Tijerina, and Rick Russell

Figures S1–S3
Tables S1–S7

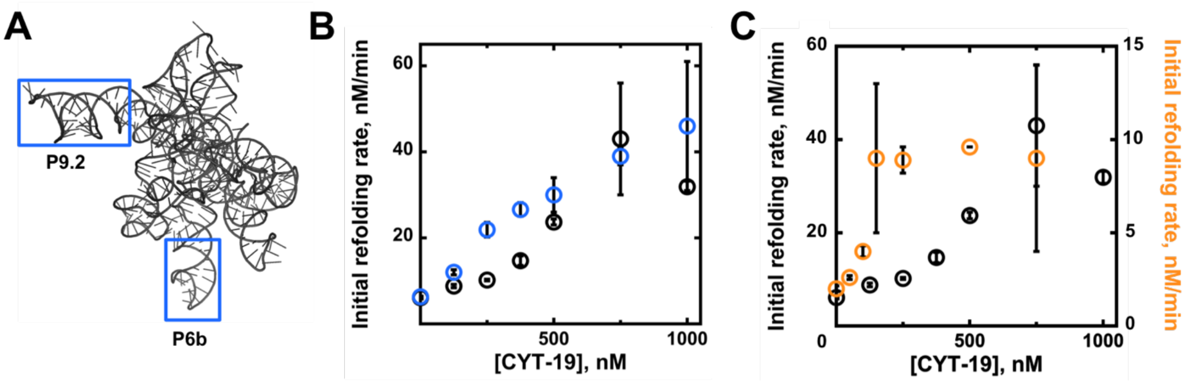

Figure S1: CYT-19-mediated refolding of misfolded wild-type and truncation mutant ribozymes

(*A*) Model of the *Tetrahymena* group I intron ribozyme with helices that are truncated in the ΔP9.2+ΔP6b mutant boxed (37) (Figure 1A). (*B*) CYT-19 concentration dependence of refolding of the misfolded wild-type (black) and ΔP9.2+ΔP6b truncation mutant (blue) ribozymes (200 nM ribozyme, 500 µM ATP-Mg^{2+}, 2 mM $Mg(OAc)_2$). Averages and standard errors from two determinations are shown. For the wild-type ribozyme, only the data collected side by side with the truncation mutant are included; these rates were consistent with the overall averages shown in Figure 1B. The spontaneous refolding rates are identical for the wild-type and mutant ribozymes, indicating unaffected stability of the misfolded conformation. If the P6b and P9.2 helices constituted productive CYT-19 interaction sites for ATP-dependent unfolding of the misfolded ribozyme, lower CYT-19-mediated refolding rates would be predicted. Such a decrease was not observed, with the truncation mutant even showing slightly greater refolding rates, perhaps because of an increased concentration of free CYT-19 available for productive interactions due to loss of the constitutively accessible surface helices that may provide a 'sink' for CYT-19 binding. (*C*) CYT-19 concentration dependence for refolding of 200 nM (black) and 50 nM (orange) misfolded wild-type ribozyme. The 200 nM ribozyme data are reproduced from panel *A* for comparison, with the data for 50 nM ribozyme collected during the same experiments. Averages and standard errors from two determinations are shown. With the lower ribozyme concentration, CYT-19-mediated refolding was faster and there was no apparent upward curvature in the CYT-19 concentration dependence. This result suggests that the upward curvature in the data with 200 nM ribozyme arose because CYT-19 binds to the RNA and is unavailable for functional interactions, producing a titration effect.

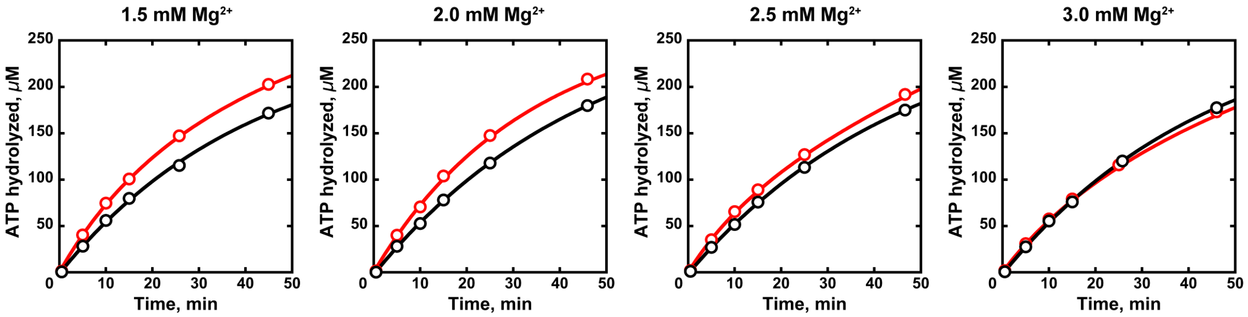

Figure S2: ATPase stimulation by the misfolded and native wild-type ribozymes at varying Mg^{2+} concentrations

The data illustrate a Mg^{2+}-dependent decrease in the ATPase stimulation by the misfolded ribozyme (red) relative to the stimulation by the native ribozyme (black). Full time course data are shown. Reactions were performed in the presence of 200 nM ribozyme, 500 nM CYT-19, 500 μM ATP-Mg^{2+}, pH 7.0.

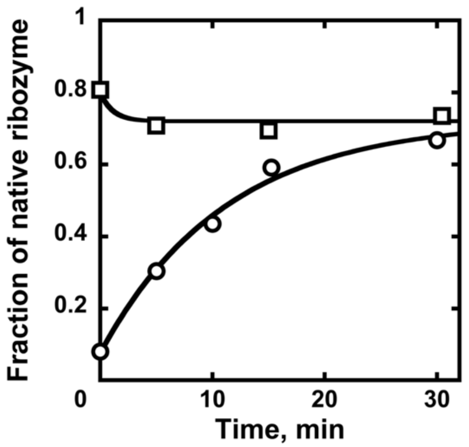

Figure S3: Unfolding of the native ribozyme by CYT-19 at 1 mM Mg^{2+}

Unfolding of the native ribozyme (squares) at 1 mM Mg^{2+} in the presence of 500 nM CYT-19, 200 nM ribozyme, and 200 μM ATP- Mg^{2+}. Refolding of the misfolded ribozyme (circles) at the same conditions is also shown, illustrating incomplete refolding due to concurrent unfolding of the native ribozyme.

Table S1: ATP concentration dependence of the refolding, ATPase rates and of ATP utilization (500 nM CYT-19, 200 nM ribozyme, 2 mM $Mg(OAc)_2$)

[ATP], µM	Initial refolding rate, µM min^{-1}	Initial ATPase rate, µM min^{-1}			ATP molecules per M ribozyme*
		Misfolded (M)	Native (N)	M-N	
0	0.0070 ± 0.0004 *(9)***				
100	0.0123 ± 0.0019 *(2)*	1.91 ± 0.15 *(6)*	1.37 ± 0.10***	0.54 ± 0.09****	100 ± 40
200	0.0159 ± 0.0019 *(4)*	3.4 ± 0.2 *(5)*	2.3 ± 0.3	1.05 ± 0.16	120 ± 30
500	0.0267 ± 0.0016 *(10)*	6.8 ± 0.3 *(10)*	4.5 ± 0.3	2.4 ± 0.3	119 ± 19
1000	0.033 ± 0.003 *(2)*	9.7 ± 0.8 *(3)*	6.5 ± 0.8	3.20 ± 0.07	124 ± 14
2000	0.036 ± 0.003 *(6)*	12.0 ± 0.7 *(7)*	9.2 ± 0.3	2.8 ± 0.7	100 ± 30
3000	0.044 *(1)*	17.1 *(1)*	12.7	4.4	121

* ATP utilization was calculated based on the difference between the initial ATPase rates measured with the misfolded and native ribozymes, divided by the difference between the refolding rates with and without CYT-19 and ATP (i.e., CYT-19 and ATP-dependent refolding rate):

$$\#\text{ATP} = \frac{\text{ATPase rate(M) - ATPase rate(N)}}{\text{Refolding rate - Refolding rate(no ATP)}}.$$

** Average and standard error, with the number of measurements indicated in parentheses.

*** Ref. 33.

**** Averages and standard errors were calculated based on the differences in ATPase stimulation observed between the misfolded and native ribozyme in each experiment.

Table S2: CYT-19 concentration dependence of the refolding, ATPase rates and of ATP utilization at 500 µM ATP-Mg^{2+} (200 nM ribozyme, 2 mM $Mg(OAc)_2$)

[CYT-19], nM	Initial refolding rate, µM min^{-1}	Initial ATPase rate, µM min^{-1}			ATP molecules per M ribozyme*
		Misfolded (M)	Native (N)	M-N	
0	0.0061 ± 0.0003 *(2)*				
125	0.0083 ± 0.0005 *(4)*	0.88 ± 0.12 *(3)*	0.76 ± 0.10	0.11 ± 0.06	50 ± 30
250	0.0101 ± 0.0004 *(4)*	1.99 ± 0.17 *(4)*	1.84 ± 0.17	0.16 ± 0.05	39 ± 12
375	0.0150 ± 0.0011 *(4)*	4.4 ± 0.3 *(5)*	3.7 ± 0.2	0.7 ± 0.2	80 ± 30
438	0.020 *(1)*	5.2 ± 0.2 *(3)*	4.5 ± 0.3	0.74 ± 0.10	53 ± 7
500	0.0267 ± 0.0016 *(10)*	6.8 ± 0.3 *(10)*	4.5 ± 0.3	2.4 ± 0.3	114 ± 18
625	0.032 *(1)*	8.7 ± 0.3 *(3)*	5.8 ± 0.2	2.9 ± 0.4	111 ± 17
750	0.039 ± 0.006 *(4)*	10.7 ± 0.7 *(4)*	6.9 ± 0.4	3.8 ± 0.5	120 ± 30
1000	0.0305 ± 0.0013 *(3)*	11.3 ± 0.8 *(2)*	7.6 ± 1.0	3.71 ± 0.19	152 ± 12

* ATP utilization was calculated based on the difference between the initial ATPase rates measured with the misfolded and native ribozymes, divided by the CYT-19 and ATP-dependent refolding rate:

$$\#ATP = \frac{\text{ATPase rate(M) - ATPase rate(N)}}{\text{Refolding rate - Refolding rate(no CYT-19)}}.$$

Table S3: CYT-19 concentration dependence of the refolding, ATPase rates and of ATP utilization at 2 mM ATP-Mg^{2+} (200 nM ribozyme, 2 mM $Mg(OAc)_2$)

[CYT-19], nM	Initial refolding rate, μM min^{-1}	Initial ATPase rate, μM min^{-1}			ATP molecules per M ribozyme
		Misfolded (M)	Native (N)	M-N	
0	0.0079 ± 0.0008 *(3)*				
250	0.015 *(2)*	5.0 ± 1.4 *(2)*	5 ± 2	0.4 ± 0.6	50 ± 90
375	0.022 ± 0.005 *(2)*	7.3 ± 0.9 *(3)*	5.4 ± 0.6	1.9 ± 1.2	140 ± 100
500	0.036 ± 0.003 *(6)*	12.0 ± 0.7 *(7)*	9.2 ± 0.3	2.8 ± 0.7	100 ± 30
625	0.042 *(1)*	15.0 ± 0.3 *(2)*	12.0 ± 0.2	3.03 ± 0.08	89 ± 3
750	0.045 ± 0.006 *(2)*	17.3 ± 0.4 *(2)*	14 ± 2	3 ± 2	90 ± 50
1000	0.056 *(1)*	27.7± 1.7 *(3)*	14.3 ± 0.4	14 ± 2	280 ± 40

Table S4: Mg^{2+} concentration dependence of ATP utilization for refolding of the WT ribozyme (500 nM CYT-19, 500 µM ATP-Mg^{2+}, 200 nM ribozyme)

$[Mg^{2+}]$, mM	Initial refolding rate, µM min^{-1}		Initial ATPase rate, µM min^{-1}			ATP molecules per M ribozyme
	- CYT-19	+ CYT-19	Misfolded (M)	Native (N)	M-N	
1.5	0.0082 *(1)*	0.032 *(1)*	7.7 *(1)*	5.8	1.9	110
2	0.0061 ± 0.0003 *(10)*	0.0267 ± 0.0016 *(10)*	6.8 ± 0.3 *(10)*	4.5 ± 0.3	2.4 ± 0.3	114 ± 18
2.5	0.005 *(1)*	0.0172 *(1)*	6.7	5.3	1.7	140

Table S5: Mg^{2+} concentration dependence of ATP utilization for refolding of the truncation mutant (ΔP9.2 + ΔP6b) ribozyme (1 µM CYT-19, 2 mM ATP-Mg^{2+}, 200 nM ribozyme)

$[Mg^{2+}]$, mM	Initial refolding rate, µM min^{-1}		Initial ATPase rate, µM min^{-1}			ATP molecules per M ribozyme
	- CYT-19	+ CYT-19	Misfolded (M)	Native (N)	M-N	
2	0.0085 ± 0.0003 *(2)*	0.041 ± 0.003 *(2)*	21 ± 3 *(2)*	9 ± 3	11.8	360 ± 20
3	0.00512 ± 0.00008 *(2)*	0.0325 ± 0.0011 *(2)*	14.7 ± 1.3 *(2)*	7.8 ± 1.9	7.0 ± 0.6	260 ± 20
4	0.0029 *(1)*	0.0144 *(1)*	17.4 *(1)*	10.4	7.0	610
5	0.00124 ± 0.00010 *(2)*	0.0074 ± 0.0002 *(2)*	15.5 ± 0.6 *(2)*	10.6 ± 0.4	4.9 ± 1.0	800 ± 160

Table S6: ATP utilization for refolding of misfolded WT and mutant ribozymes (500 nM CYT-19, 2 mM ATP-Mg^{2+}, 200 nM ribozyme, 5 mM Mg^{2+})

Variant	Initial **refolding rate, μM min^{-1}**		**Initial ATPase rate, μM min^{-1}**			**ATP molecules per M ribozyme**
	**- CYT-19**	**+ CYT-19**	**Misfolded (M)**	**Native (N)**	**M-N**	
WT	0.0012 ± 0.0002 *(3)*	0.0052 ± 0.0014 *(3)*	10.2 ± 1.3 *(3)*	7.4 ± 0.9	2.9 ± 0.5	700 ± 300
ΔL9/P5	0.0034 ± 0.0002 *(2)*	0.017 ± 0.003 *(2)*	10.7 ± 1.4 *(2)*	8.5 ± 1.7	2.2 ± 0.3	160 ± 40
ΔP13	0.0041 ± 0.0013 *(5)*	0.033 ± 0.006 *(5)*	12.9 ± 1.2 *(2)*	10.45 ± 0.15	2.1 ± 1.1	70 ± 40

Table S7: Background ATPase rates measured in the absence of added RNA (500 nM CYT-19, 2 mM $Mg(OAc)_2$)

[ATP], µM	Initial ATPase rate, $\mu M\ min^{-1}$
100	0.05 ± 0.2 *(8)* *
250	0.22 *(1)*
500	0.30 *(1)*
1000	0.41 *(1)*
2000	0.22 ± 0.16 *(3)*
4000	0.63 *(1)*

* Ref. 33.
